# Timed Up and Go (TUG) Predicts Changes in the Kihon Checklist After Six Months of Continuous Participation in "Kayoinoba" (Community Gathering Places)

**DOI:** 10.7759/cureus.83394

**Published:** 2025-05-03

**Authors:** Takashi Nasu, Keisuke Kubota, Hiroo Furusawa, Takaya Abe, Toyohiro Hamaguchi, Naohiko Kanemura, Yoshinori Kitabatake, Toshiyuki Yoshida, Takahito Nakamura, Yuji Matsumura

**Affiliations:** 1 Rehabilitation, Koshigaya Seiwa Hospital, Koshigaya, JPN; 2 Health Sciences, Hirosaki University Graduate School, Hirosaki, JPN; 3 Rehabilitation, Koshigaya Rehabilitation Liaison Council, Koshigaya, JPN; 4 Physical Therapy, School of Health and Social Services, Saitama Prefectural University, Koshigaya, JPN; 5 Research Development Center, Saitama Prefectural University, Koshigaya, JPN; 6 Rehabilitation, Rehabilitation Amakusa Hospital, Koshigaya, JPN; 7 Rehabilitation, Saitama Prefectural University, Koshigaya, JPN; 8 Health Development, Saitama Prefectural University, Koshigaya, JPN; 9 Health Sciences, Saitama Prefectural University, Koshigaya, JPN; 10 Thoracic Surgery, Koshigaya Seiwa Hospital, Koshigaya, JPN

**Keywords:** community gathering, frailty, kayoinoba, kihon checklist, nursing care prevention, timed up and go

## Abstract

Introduction: This study aimed to predict frailty in older adults participating in community-based gatherings in a local city over a six-month period.

Methods: In total, 257 older community members participated in this study. The Kihon Checklist (KCL) was used to assess life function. The KCL consists of 25 items, each scored as 0 or 1 point, resulting in a total score ranging from 0 to 25. Higher scores indicate a greater risk of requiring long-term care. The items cover six domains: physical function, nutritional status, oral function, social engagement, cognitive function, and depressive mood. For motor function assessment, the Timed Up and Go (TUG), one-leg standing, and 30-second chair-stand (CS-30) tests were used.

Results: Multiple regression analysis was used to investigate the effects of age, gender, and motor function on the KCL after six months. The regression formula was set as: KCL after six months (points) = first TUG (seconds) * 0.29 + first KCL (points) * 0.67 - 0.99. The model demonstrated moderate to strong predictive accuracy (adjusted R² = 0.53, root mean squared error (RMSE) = 1.74, mean absolute error (MAE) = 1.34).

Conclusion: Our results showed that TUG could serve as an indicator for older adults participating in community-based gatherings in a local city, implementing our program, and improving the KCL.

## Introduction

In Japan, frailty prevention initiatives were spotlighted alongside the 2005 revision of the long-term care insurance system, which now prioritizes preventing the need for nursing care [1]. Starting in 2011, various initiatives were introduced to create a community-based care system, allowing senior citizens to continue living in their communities and maintain their preferred lifestyle until their final days of life [2]. In this study, we use the term "Kayoinoba" to refer to community-based gathering places for older adults, which are part of Japan’s nationwide frailty prevention initiatives. These gatherings are facilitated by local governments and are characterized by regular group exercise and social participation. 

Among these, certain policies aimed at preventing frailty and the need for nursing care to encourage participation of senior citizens in their communities, including organizing community gatherings (Kayoinoba) for older adults, focusing on local residents [3]. 

These Kayoinoba allow senior citizens to participate with the support of experts and other helpers, with the aim of prolonging the time that senior citizens can live without nursing care. Kayoinoba is a national initiative promoted by the Japanese Ministry of Health, Labour, and Welfare to prevent frailty and the necessity for long-term care. While the specific activities vary across municipalities, Kayoinoba programs generally include structured exercises, oral health training, cognitive activities, and opportunities for social engagement. These programs are designed to encourage regular participation and comprehensive health maintenance among older adults. Although there is no nationwide standardized format, Kayoinoba programs typically share the common goal of promoting both social interaction and physical health among senior citizens. While activities differ by municipality, most programs incorporate structured physical exercises along with social engagement activities. In certain regions, Kayoinaba primarily functions as a social space, while in others, such as Koshigaya City, it is implemented as an exercise-focused program. The implementation in Koshigaya City, emphasizing a structured exercise-based format, reflects one of the most common patterns of Kayoinoba throughout Japan. Currently, many of these Kayoinoba have been established in municipalities nationwide, and the content of these initiatives has been researched and reported. Research findings indicate that senior citizens participating in these Kayoinoba exhibited a subjective sense of good health [4], maintenance of physical function [5], increased participation in society [6], and reduced risk of requiring support or nursing care [7,8]. Consequently, it is believed that senior citizens engaged in these Kayoinoba can maintain or enhance their mental and physical health and quality of life. Koshigaya City in Saitama Prefecture, Japan, has been developing a Kayoinoba program for senior citizens, and findings from the National Institute of Population and Social Security Research suggest that continued participation improved the mental and physical health and quality of life of senior citizens [9]. 

However, continued attendance is hindered by the monotony of activity content and the decline in health status with the increasing age of participants [10]. A previous study conducted by our affiliated group reported that approximately 30% of participants discontinued their participation [11]. It is noted that motivation and self-efficacy are crucial in sustaining exercise involvement [12], and setting goals is one effective countermeasure [11]. Therefore, maintaining participant motivation and encouraging continuous engagement are essential for the long-term operation of the program. Establishing benchmark values for initial motor function assessments (such as Timed Up and Go (TUG) or 30-second chair-stand (CS-30)) could aid in setting personalized goals for participants and promoting continued participation in Kayoinoba. If it becomes feasible to predict caregiving risk resulting from continuous participation in the Kayoinoba based on the assessment of care risk and motor function evaluation obtained through questionnaires at initial enrollment, it would be possible to set goals for participants and encourage their active involvement. Specifically, walking ability, as represented by motor function [13], relates to a wide range of daily living activities, and evidence shows that participation rates are higher when Kayoinoba is located close to one's home [4]. Recognizing the characteristics of such settings, establishing benchmark values for motor function could be highly advantageous. Therefore, the goal of this study was to develop a predictive model capable of forecasting both caregiving risk and physical function outcomes after six months of participation in the Kayoinoba, based on the initial assessment of care risk and motor function evaluation. We focused on the predictive utility of simple physical performance tests and the Kihon Checklist (KCL), which is widely used to assess frailty status in older adults. This evaluation could provide valuable insights for both Kayoinoba operators and participants.

Although Kayoinoba is promoted nationwide as part of Japan’s frailty prevention strategy, there are no strict national standards for implementation. Each municipality possesses the autonomy to design its own Kayoinoba program according to local needs and resources. While many programs include physical exercise, the degree of emphasis on exercise and social activities varies by location. The core concept, however, remains centered on providing a community space to support both physical and social well-being. Kayoinoba was originally conceived as a community-based gathering place to foster interaction among older adults, with specific activities varying by region. In Koshigaya City, it is implemented as a structured exercise program, representing the most common form observed nationwide. Therefore, this study emphasizes both the social and physical activity components of Kayoinoba, highlighting their significance in preventing frailty and improving motor function among older adults.

## Materials and methods

Study design and participants

Koshigaya City launches the Kayoinoba program annually, and we assess participants at the program's start. Since 2016, first-time participants have undergone physical aptitude tests at 41 Kayoinoba locations in Koshigaya City, with follow-up tests conducted six months later. The activities at Kayoinoba include four consistent types of exercises: (i) Dynamic stretching as a warm-up (10 minutes). This component typically includes rhythmic upper limb movements and trunk exercises designed to enhance shoulder mobility and scapular motion. These movements are performed in a standing position and aim to gradually increase circulation and range of motion in preparation for the main exercises. (ii) Koshigaya Rakunobi exercise, which consists of resistance exercises for the upper and lower limbs using therabands (10 minutes). Specific exercises include shoulder flexion and elbow extension in a seated position, knee extension exercises also in a seated position, and hip abduction exercises performed while standing. These movements are designed to improve muscular strength and joint mobility and are adjusted to match the physical abilities of older participants. (iii) Rakusho exercise, which involves rhythmic body movements performed in coordination with singing to enhance oral and respiratory function (10 minutes). Specific movements include knee extension exercises performed in a seated position and hip abduction exercises performed while standing. These activities synchronize with singing and aim to integrate cognitive, respiratory, and motor functions in an engaging and socially interactive format. (iv) Static stretching for cool-down (five minutes). This includes static stretches targeting the shoulders and lower limbs, as well as deep breathing exercises to promote relaxation and gradual recovery after physical activity. Static stretching involves maintaining a stretch position without movement, typically held for 10-20 seconds to promote flexibility and relaxation.

This was a retrospective cohort study measuring the motor function of participants in the Kayoinoba program using the KCL at initial assessment (start of participation) and six-month follow-up. The software G*Power (Heinrich-Heine-Universität Düsseldorf, Düsseldorf, Germany) was used to determine the sample size. The settings were as follows: effect size f², α error = 0.05, and power (1-β) = 0.95. Consequently, the required sample size was 146.

**Figure 1:**
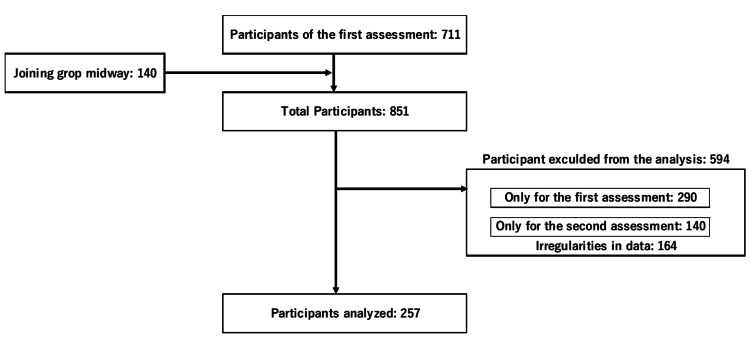
Flowchart showing the selection of participants for analysis

The study identified participants through resident leaders who helped establish the Kayoinoba by distributing flyers and utilizing community bulletins. Eligible participants were selected from regular attendees at their local Kayoinoba. Of the 851 participants surveyed at their first session, 257 attended the second evaluation and did not lack any data; thus, they were included in the analysis. Regarding missing values, we examined which survey items might influence changes in the KCL, an indicator of frailty. Therefore, items with even a single missing value were excluded using the list-wise deletion method (Figure 1). The evaluation criteria included the KCL and the physical aptitude test. The KCL is a self-evaluation survey comprising 25 questions regarding lifestyle and mental and physical function, where participants answer either yes or no to each question. It serves as a preliminary assessment of frailty in frailty diagnostic guides, and its validity as an evaluation method has been verified [14]. In Japan, the KCL is widely used for screening caregiving risk and is a valuable index for identifying frailty in older adults [15,16].

KCL and physical aptitude test

KCL questions include five questions about instrumental activities of daily living (IADL), five about physical function, two about nutritional status, three about oral function, two about outdoor activities, three about cognitive function, and five about depressed mood, totaling seven evaluation items. Each question is scored as either 0 (no problem) or 1 (problem present), resulting in a total score ranging from 0 to 25 points. Higher scores indicate a greater risk of frailty. The score for each domain is calculated by summing the responses within that domain, allowing for a more detailed assessment of functional, nutritional, cognitive, and psychological risks. The area under the receiver operating characteristic curve for assessing frailty status with the KCL was 0.81 for pre-frailty and 0.92 for frailty. When the KCL cutoff scores were set at 4 points for distinguishing pre-frailty and 8 points for identifying frailty, the reported sensitivity and specificity were 70.3% and 78.3%, respectively, for detecting pre-frailty, and 89.5% and 80.7%, respectively, for detecting frailty [16].

The TUG test, which measures the time it takes participants to stand up from a chair, turn over a marker three meters away, and then return to sitting in the chair, was used to assess motor function. It is widely recognized as an indicator for evaluating lower limb muscle strength and mobility [17]. The shorter the time required to perform the task, the higher the evaluation score. The TUG has demonstrated reliability for use in adults, children, and patients with various illnesses [18]. Participants were instructed to perform the TUG test at their maximum possible speed. The measurements were taken in a community center with hard flooring, such as wooden floors; participants were instructed to wear indoor shoes with heels or to go barefoot during the measurements. While performing the TUG, participants were allowed to use their usual walking aids, such as canes or orthoses, if these devices were part of their daily routine. The use of familiar assistive devices was allowed to ensure safety and to reflect the participants’ actual functional mobility.

The balance test was used to evaluate one-leg standing time. For the one-leg standing test, participants were instructed to perform the test on their dominant leg, and a single trial was conducted. The time they could maintain the position without support (up to 120 seconds) was recorded. Due to time constraints in the assessment protocol, repeated trials were not performed. Participants were instructed to raise their dominant leg without allowing it to touch the ground or the supporting leg, while placing their hands on their hips and keeping their eyes open. The time maintained in this posture (up to 120 seconds) was measured to evaluate their balance ability. The longer participants could balance, the higher their evaluation score. The one-leg stand test has been reported to be highly reliable [19]. For this study, the evaluation focused on the right lower limb. The CS-30 assesses how many times a participant can stand up from a chair within 30 seconds and is an easily administered test of lower limb strength [20]. The CS-30 was conducted using a chair with a seat height of 40 cm, which was standardized across all measurement sites. The same type of chair was used at each location to ensure consistency in test conditions. The more times a participant could stand up from the chair within the allotted time, the higher their evaluation score. The CS-30 has been reported to be highly reliable [21]. These assessments were selected based on the understanding that basic characteristics such as age and gender influence the progression of frailty.

For motor function assessments, the TUG test was included as an index of walking ability and the CS-30 test as a measure of lower limb muscle strength, given the relationship between sarcopenia and frailty. Additionally, the one-legged stance test was included as an indicator of balance function, as balance is also associated with frailty. These evaluations were conducted by staff from the Koshigaya Rehabilitation Liaison Council, responsible for nursing-care preventive therapy in Koshigaya City. Evaluation methods were outlined in a manual, and staff members conducting the evaluations had ample time to study and practice them. The Kayoinoba program is implemented across Koshigaya City under the National Institute of Population and Social Security Research. This study was conducted in accordance with the Enhancing the Quality and Transparency of Health Research (EQUATOR) guidelines.

Statistical analysis

Statistical analysis was performed using the numerical analysis software MATLAB R2022a (MathWorks Inc.; Natick, USA). The total KCL score was treated as a continuous variable, based on the accumulation of deficits model [22,23], which conceptualizes frailty as a continuous construct. This approach aligns with previous studies [16], which also analyzed the KCL score as a continuous outcome in regression analyses. To assess the influence of each factor on the KCL, the score after six months was used as the objective variable. Age, sex, initial KCL, initial TUG, one-leg standing time, and CS-30 scores served as explanatory variables, and multiple regression analysis using a stepwise method was performed. Multicollinearity was evaluated using the variance inflation factor (VIF), with a threshold of five or higher. For assessing the predictive accuracy of the regression model, the root mean squared error (RMSE) and mean absolute error (MAE) were calculated. RMSE measures the average magnitude of the error, giving more weight to larger errors, while MAE represents the average absolute difference between predicted and actual values, providing an intuitive sense of error size. To examine the relationships between TUG and each functional measure, Spearman's rank correlation coefficients were calculated using initial TUG and age, one-leg standing time, CS-30 scores, and total KCL scores. Statistical significance was set at a risk rate of less than 5%. 

Ethical considerations

This study was conducted in accordance with the principles of the Declaration of Helsinki. Prior to the first and second evaluations, participants were given a thorough verbal explanation, and their consent was obtained. This study was approved by the Koshigaya Seiwa Hospital Ethics Committee (approval number: 2023-02).

## Results

A total of 851 people participated in the Kayoinoba program. Of these, 583 were excluded from the study (290 had data only for the first period (dropped out), 140 had data only for the second period (joined midway through the study period), and 164 had incomplete data. Thus, 257 (257/851) were finally included in the study (Figure 1).

The mean age of the participants was 74.8 ± 6.3 years, comprising 39 men (mean age: 76.9 ± 5.2 years) and 218 women (mean age: 74.5 ± 6.4 years) (Table 1). Significant improvements were observed in the total KCL score, outdoor activity frequency, and CS-30 after six months. Results from the multiple regression analysis (Table 2) revealed that the initial KCL and initial TUG significantly predicted the KCL score after six months. All variables in the model had a VIF below five.

**Table 1:** Basic information of Kayoinoba participants (n=257) Values are presented as mean ± standard deviation for age and count for sex.

Variable	Value
Age, years	74.8 ± 6.3
Sex, n (men/women)	39/218
Age by sex, years	Men: 76.9 ± 5.2, women: 74.5 ± 6.4

**Table 2:** Multiple regression analysis using KCL after six months as objective variable B: Unstandardized regression coefficient; KCL: Kihon Checklist; TUG: Timed Up and Go; CS-30: 30-second chair-stand; VIF: Variance inflation factor.

Model Summary						
Multiple R²: 0.539	Adjusted R²: 0.528					
F-statistic: 49.11	p-value: <0.01					
Coefficients (Variables Included in the Equation)						
Variable	B	Std. Error	t	p-value	95% Confidence Interval for B	VIF
Constant	-0.99	0.59	-1.69	0.09	-7.12 to 1.22	-
TUG	0.29	0.09	3.28	<0.01	0.04 to 0.44	1.47
First KCL	0.67	0.05	14.9	<0.01	0.57 to 0.76	1.3
Variables Not Included in the Equation						
Variable	B	Std. Error	t	p-value	95% Confidence Interval for B	VIF
CS-30	-0.01	0.59	-1.39	0.164	-7.12 to 1.22	1.48
Sex	-0.51	0.39	1.32	0.19	-1.27 to 0.08	1.02
Age	0.03	0.03	14.9	<0.01	-0.02 to 0.76	1.38
One-leg stand	-0.01	0.01	-0.21	0.83	-0.01 to 0.01	1.31

The regression formula was set as: KCL after six months (points) = first TUG (seconds) * 0.29 + first KCL (points) * 0.67 - 0.99. The coefficient of determination for the model was 0.53 with adjusted R². Additionally, RMSE was 1.74 and MAE was 1.34, suggesting acceptable predictive accuracy. A correlation between the TUG score and the total KCL score was observed (Table 3), and moderate correlations were also found for one-leg standing time, CS-30, and age.

**Table 3:** Correlation coefficient between TUG and each factor TUG: Timed Up and Go; KCL: Kihon Checklist; CS-30: 30-second chair-stand; * denotes p<0.05.

Category	Factor	Correlation coefficient	p-value
Basic information	Age	0.36	<0.01*
Motor function	One leg stand	-0.39	<0.01*
	CS-30	-0.41	<0.01*
KCL	Total score	0.12	0.04*

## Discussion

The purpose of this study was to develop a predictive model capable of forecasting both caregiving risk and physical function outcomes six months after participation in the Kayoinoba program, based on the initial assessment of care risk and motor function evaluation. The results showed that the TUG and the first KCL influenced the six-month KCL. According to this prediction formula, for example, participants with a KCL score of 3, the lower limit of normal, and a TUG of 6.8 seconds or less may be able to maintain or improve their KCL. Additionally, a participant with a basic checklist score of 7, the lower limit of pre-frailty, and a TUG of 11.4 seconds or less may maintain or improve their KCL. This suggests that the initial TUG score may serve as an effective indicator reflecting caregiving risk.

The results of this study showed that the TUG score correlated with the risk of nursing care after six months, as assessed by the KCL. TUG is used as an evaluation index for balance and walking ability [24,25]. Walking speed is related to age and quadriceps muscle strength, as measured by the CS-30 [26,27]. Therefore, the TUG is an evaluation of motor function that reflects physical function as a whole. To participate in the Kayoinoba program, participants need to leave their homes, and in many cases, this requires them to navigate steps and stairways. In addition, the Kayoinoba program in Koshigaya City requires participants to assume various standing positions [9], necessitating a certain level of balance. Due to these factors, participants must possess a minimum level of physical function to continue participating in the program. The results of this study indicate that participants must maintain physical function and ongoing participation for us to continue our programs; this is reflected in the TUG results indicating overall physical function. However, the regression equation derived from this study indicates that the KCL after six months is influenced by the TUG results. While the Koshigaya City Kayoinoba program has proven effective for individuals with pre-frailty, its impact on individuals with frailty is somewhat limited, as supported by previous studies. The regression equation highlights that, to maintain a KCL score of 8 (the lower limit of pre-frailty after six months), the initial TUG must be faster than 12.5 seconds. Participants with a TUG time greater than 12 seconds have a higher risk of declining IADL and a greater chance of reaching a time of 13.5 seconds or more [28]. Furthermore, factors affecting the decline in the KCL score include a lack of sufficient walking and balancing ability to participate in the Kayoinoba program; therefore, it is believed that overall physical function may have declined for these individuals with frailty. TUG showed a positive correlation with walking speed [28]; therefore, we theorize that TUG results may depend on walking speed. Additionally, walking speed reflects overall physical condition and is related to a future decline in function [29,30]. Consequently, we conclude that the longer the TUG time (which reflects general motor function), the greater the risk of decline in physical function in the future. Participants with longer TUG times may require more intensive interventions. This study shows that participants with a certain level of TUG performance are likely to have adequate physical function to continue attending the Kayoinoba program, and their six-month changes in KCL can be predicted from baseline TUG results. This suggests that TUG may serve as a useful predictor of caregiving risk over time.

In recent years, community-based interventions to prevent frailty have gained global attention. For example, in Europe, initiatives such as "active ageing" [31] and "social prescribing" [32] in the United Kingdom have focused on promoting social participation among older adults to reduce frailty-related risks. Similarly, the World Health Organization (WHO) has emphasized the importance of integrated community care and active aging strategies worldwide. These trends align with the objectives of the Kayoinoba program in Japan, suggesting that findings from this study may have relevance beyond national borders and could inform the development of similar programs internationally. The predictive model used in this study could be valuable as an indicator for identifying these individuals. The coefficient of determination (adjusted R²) in this study's analysis was 0.53. In the multiple regression analysis of this study, the adjusted R² was 0.53, indicating a moderate to strong level of explanatory power for predicting the KCL score. This value falls within the range considered acceptable for predictive accuracy in the fields of social and medical sciences [33]. Given that the model used only two simple indicators - TUG and the initial KCL score - to explain over half of the variance, the findings suggest that the model is not only statistically valid but also clinically useful as a frailty prediction tool. To further evaluate the model’s predictive accuracy, the RMSE and MAE were calculated. The obtained values (RMSE = 1.74, MAE = 1.34) fall within an acceptable range when compared to the scale of the KCL score, indicating that the model has sufficient predictive accuracy. Furthermore, the adjusted R² value of 0.53 is considered moderate to strong in social and medical sciences, supporting the model’s validity as a practical tool for clinical application. Although this study did not include a control group of non-participants, the observed improvements in KCL scores suggest that continued participation in Kayoinoba may contribute to a reduced risk of requiring long-term care. Future research should investigate how caregiving risk differs between those who participate in Kayoinoba and those who do not. Quantifying this difference would not only strengthen the scientific basis of community-based interventions but could also help motivate older adults by providing tangible evidence of the benefits of participation. In addition, the RMSE of 1.74 and MAE of 1.34 further support the model's predictive validity, indicating that the formula has sufficient accuracy for use in clinical and community settings. 

One of the notable limitations of the Kayoinoba program is the dropout rate between the initial and follow-up evaluations. Several factors may have contributed to this, including decreased motivation, transportation difficulties, and health-related issues. Additionally, since the program included only a single follow-up evaluation day, it is expected that some participants may have been unable to attend due to scheduling conflicts or health conditions. To address this issue, we believe that introducing individualized goal setting based on the initial evaluation and providing regular feedback may enhance participant engagement and program adherence. In this context, the present study may play an important role by helping establish meaningful goals for continued participation. Although this study focused on the Kayoinoba program in Koshigaya City, similar challenges related to physical access and attendance may occur in other municipalities. The extent of these issues may vary depending on the frailty status of participants and geographical factors such as distance to the venue or local terrain. Therefore, regional context should be taken into account when interpreting and applying these findings.

This study demonstrated that the six-month change in KCL can be predicted based on the baseline TUG performance, suggesting that TUG may serve as a practical indicator for estimating caregiving risk over time. There are many advantages to participating in the Kayoinoba program. For physically frail individuals, commuting to the Kayoinoba can help improve physical function and increase physical activity [34,35]. However, while the level of physical activity is related to physical frailty, it is not related to cognitive or social frailty [36]. Therefore, simply increasing the level of physical activity is not enough to prevent frailty as a whole. Participating in the Kayoinoba may encourage participants to join other groups as well, which may be effective in preventing cognitive decline and mental and social weakness [8,37,38].

Among senior citizens who do not exhibit physical weakness but may exhibit social weakness, the risk of the onset of physical weakness is about four times greater [39]. Therefore, reducing social limitations on senior citizens is vital for preventing physical frailty. Six months of culture, arts, and science programs aimed at senior citizens have been demonstrated to improve physical function, subjective sense of well-being, and mental acuity [5]. Hence, it is believed that regular social engagement can have a holistic positive effect on health in older adults.

Reports from Kayoinoba programs in other municipalities that mainly focused on exercise showed that after one year, walking speed, TUG, time for standing up five times from a chair, and hand grip strength all improved [35,40]. Our study also examined a program that primarily focused on exercise. While improvements in physical function were not observed (possibly due to the shorter study period), improvements were noted in the KCL items related to depressed mood and self-isolation. Depression in older people has been linked to reduced participation in society [9], including social activities and interaction with friends [41,42]. Kayoinoba programs provide exercise opportunities and promote relationships and communication among participants based on shared hobbies and interests. Commuting to a Kayoinoba program encourages communication among participants and, therefore, may help prevent mental and emotional decline. It has been shown that participants experienced a greater subjective sense of well-being when exercising in groups rather than alone, which led to decreased levels of depressed mood [40,41]. Consequently, the Koshigaya City Kayoinoba program is thought to effectively improve physical function and holistically prevent frailty. Participants need a minimum level of mobility to achieve these comprehensive effects of Kayoinoba. Although this study explored the link between physical function and continued participation in Kayoinoba, it's crucial to note that Kayoinoba was not originally designed as a formal medical intervention. Instead, it serves as a community-based initiative that encourages social interaction and light physical activity among older adults. The study evaluated its potential effects through observational analysis rather than as a prescriptive intervention program. Furthermore, while this research focused on how physical function affects outcomes such as KCL scores, we acknowledge that mental health factors, such as motivation, social isolation, and depression, may also contribute to physical decline. The relationship is likely bidirectional, and future research should more closely examine how psychological well-being impacts physical outcomes in older populations. Although participation in Kayoinoba is not formally restricted based on physical abilities, regular attendance often requires a certain level of mobility due to the venues' physical structures. Thus, individuals with limited mobility may be less likely to participate or continue attending. Moreover, while this study emphasized the role of physical engagement on mental health, the reverse relationship - where psychological conditions like depression contribute to physical decline - should also be investigated in future research. In this model, the KCL offers a multidimensional view of frailty, including physical, cognitive, psychological, and nutritional domains, based on self-report. However, it may not fully reflect objective physical performance. The TUG test, on the other hand, is a quick and reliable objective measure of motor function. By integrating these two indicators, the present model captures both subjective and objective perspectives, encompassing both physical and psychosocial aspects. Given that both tools are feasible in group-based community programs such as Kayoinoba, the model offers not only strong predictive accuracy but also high practicality. These characteristics underscore the novelty and potential utility of this study’s predictive approach.

Limitations

Our study has some limitations. First, this study targeted participants who regularly commuted to the Kayoinoba program, but it did not measure their attendance frequency. Regular participation is believed to be crucial for the effectiveness of the Kayoinoba program [5]. Thus, variations in effectiveness among participants may depend on attendance frequency, suggesting that future studies should address this aspect. Second, we assessed whether each organization could maintain its activities based on government data during the COVID-19 pandemic. We collaborated with the government to exclude from the survey any groups that were unable to continue their activities within six months of establishment. Therefore, we believe that applying the results of this study today poses no significant issues. However, it remains uncertain whether the effects of the COVID-19 pandemic have been entirely eliminated. Third, although fundamental characteristics such as annual income and educational background are thought to influence frailty, they were not explored in this study. Fourth, in this study, we measured TUG without assessing walking speed due to time constraints in motor function evaluations and space limitations at the Kayoinoba venues. However, since walking speed is also recognized as an indicator of sarcopenia [42], its inclusion could provide more valuable insights. Fifth, grip strength was not measured in this study, which precluded its use as an explanatory variable. However, as grip strength is an indicator of sarcopenia [42] and has been reported as a predictor of frailty [41], we believe it should be measured in future studies. Sixth, cognitive function tests were not conducted on participants in this study. Although the relationship between cognitive function and frailty has been documented [43,44], we were unable to perform such tests due to time constraints. Seventh, it should be noted that this study analyzed only participants who continued attending the Kayoinoba program. In a previous study, we also examined individuals who dropped out and found that their TUG times were, on average, faster than those of participants who remained [9]. Therefore, we believe that trends in TUG may differ between continuers and dropouts when predicting changes in KCL, warranting further studies to explore this difference in more detail. This presents a challenge for future research.

## Conclusions

This study demonstrated that a certain level of physical function, as measured by the TUG test, is important for older adults to continue participating in Kayoinoba programs and to maintain or improve their caregiving risk status over six months, as assessed by the KCL. The findings suggest that baseline TUG performance can serve as a practical and objective indicator for predicting six-month changes in frailty risk in community-based programs. Because both the KCL and TUG are feasible and easily administered in group settings like Kayoinoba, the predictive model developed in this study offers high practicality as well as strong predictive accuracy. Furthermore, while this study focused mainly on physical function, it recognizes that psychological factors such as motivation and social participation also interact closely with physical outcomes. Future research should further investigate the bidirectional relationship between mental health and physical function to optimize frailty prevention strategies in community-based settings.
